# Mitral repair on the edge: a case report of redo transcatheter mitral edge-to-edge repair for recurrent regurgitation in Complex lateral jet anatomy

**DOI:** 10.1093/ehjcr/ytaf551

**Published:** 2025-10-22

**Authors:** Christoph Pauschinger, Benedikt Koell, Lara Waldschmidt, Niklas Schofer, Daniel Kalbacher

**Affiliations:** Department of General and Interventional Cardiology, University Heart and Vascular Center Hamburg, University Medical Center Hamburg-Eppendorf Hamburg, Martinistrasse. 52, Hamburg D-20246, Germany; German Center for Cardiovascular Research, Partner Site Hamburg/Luebeck/Kiel, Germany; Department of General and Interventional Cardiology, University Heart and Vascular Center Hamburg, University Medical Center Hamburg-Eppendorf Hamburg, Martinistrasse. 52, Hamburg D-20246, Germany; German Center for Cardiovascular Research, Partner Site Hamburg/Luebeck/Kiel, Germany; Department of General and Interventional Cardiology, University Heart and Vascular Center Hamburg, University Medical Center Hamburg-Eppendorf Hamburg, Martinistrasse. 52, Hamburg D-20246, Germany; German Center for Cardiovascular Research, Partner Site Hamburg/Luebeck/Kiel, Germany; Department of General and Interventional Cardiology, University Heart and Vascular Center Hamburg, University Medical Center Hamburg-Eppendorf Hamburg, Martinistrasse. 52, Hamburg D-20246, Germany; German Center for Cardiovascular Research, Partner Site Hamburg/Luebeck/Kiel, Germany; Department of General and Interventional Cardiology, University Heart and Vascular Center Hamburg, University Medical Center Hamburg-Eppendorf Hamburg, Martinistrasse. 52, Hamburg D-20246, Germany; German Center for Cardiovascular Research, Partner Site Hamburg/Luebeck/Kiel, Germany

**Keywords:** Degenerative mitral regurgitation, Recurrent MR, Redo M-TEER, Complex anatomy, Case report

## Abstract

**Background:**

Degenerative mitral regurgitation (DMR) is associated with substantial morbidity and mortality if left untreated. While surgical repair remains the gold standard, mitral transcatheter edge-to-edge repair (M-TEER) is an established alternative for patients at high surgical risk. However, recurrent MR after initial M-TEER remains a therapeutic challenge and is associated with poor outcomes.

**Case summary:**

A 77-year-old patient presented with recurrent heart failure symptoms due to MR after prior CARILLON^®^ device implantation for secondary MR at an external hospital in September 2019. Transoesophageal echocardiography revealed degenerative mitral valve disease characterized by a complex combination of posterior leaflet prolapse and anterior leaflet flail, resulting in a severe eccentric regurgitant jet.

The patient underwent successful M-TEER with the MitraClip® system in December 2021. In June 2023, the patient was re-hospitalized for recurrent heart failure symptoms. Imaging revealed severe recurrent MR originating lateral to the implanted device. A redo M-TEER was performed using the PASCAL® system, requiring careful navigation through a narrow residual lateral orifice. The procedure resulted in a durable reduction to mild MR with sustained clinical stability at 18-month follow-up.

**Discussion:**

This case illustrates the anatomical challenges of DMR and demonstrates the feasibility and durability of redo M-TEER in high surgical risk patients with recurrent MR. It emphasizes the importance of individualized anatomical assessment and tailored device selection in complex valve anatomies.

Learning points

- Redo M-TEER procedure is a feasible and effective option for treating severe recurrent mitral regurgitation in patients with degenerative mitral valve disease, even in the setting of complex anatomy.
- In carefully selected high-risk patients, redo M-TEER can provide durable MR reduction and improve patient-centred outcomes.

## Introduction

Degenerative mitral regurgitation (DMR) is one of the most prevalent valvular heart diseases and is associated with significant morbidity and mortality. If left untreated, mitral regurgitation (MR) can lead to progressive left ventricular remodelling, which in turn exacerbates MR, creating a vicious cycle.^1^

While surgical mitral valve repair remains the gold standard of treatment,^2,3^ the EVEREST II trial demonstrated that mitral transcatheter mitral edge-to-edge repair (M-TEER) is a feasible and safe alternative for patients with severe MR who are at high surgical risk.^4^ Notably, 5-year follow-up data showed similar mortality rates between M-TEER and surgery, although MR reduction was less durable in the percutaneous group.^5^ Three randomized controlled trials, REPAIR MR (ClinicalTrials.gov: NCT04198870), PRIMARY (ClinicalTrials.gov: NCT05051033), and MITRA-HR (ClinicalTrials.gov: NCT03271762), with the aim to compare M-TEER to surgical mitral valve repair, are currently enrolling patients with DMR. Since the initial introduction of M-TEER, clinical experience and procedural techniques have advanced substantially.^6^ As a result, new challenges—such as recurrent MR following initial M-TEER—have come to the forefront.^7^

To our knowledge, this is the first case report describing a redo M-TEER for recurrent DMR caused by residual prolapse following prior implantation of a transcatheter annuloplasty device.

## Summary figure

**Table:** 

Chronology	Treatment
09/2019	CARILLION® device implantation for presumed secondary MR at an external hospital
11/2021	Presentation at local hospital for acute heart failure. Experiencing severe dyspnoea (NYHA IV) and oedema. Decongestion due to i.v. diuretics. Transoesophageal echocardiography revealed severe DMR.
one week	Transfer to our hospital for a therapy evaluation for severe mitral regurgitation using a heart team approach.
Day 0	M-TEER procedure for DMR using one NTw device of the MitraClip system® (Abbott) with optimal post-procedural result. Reduction of severity from severe (4+) to trace/mild (0/1+)
21 months	Ambulatory follow-up in our structural heart clinic experiencing relapsing dyspnoea (NYHA III) due to recurrent severe MR with a severe eccentric regurgitation jet.
22 months	Redo M-TEER procedure for recurrent DMR using one PASCAL Ace precision device ® (Edwards). Reduction of severity from severe (4+) to mild (1+).
40 months/18 months after 2nd procedure	Follow-up experiencing stable dyspnoea (NYHA II), no oedema and no history of hospitalization due to congestive heart failure after second procedure. Echocardiography shows stable post-procedural result with mild (+1) residual MR after redo M-TEER.

Abbreviations: DMR: degenerative mitral regurgitation; MR: mitral regurgitation; M-TEER: mitral transcatheter edge-to-edge repair; NYHA: New York Heart Association

## Case report

A 77-year-old patient with preserved left ventricular ejection fraction was admitted to an external hospital for acute congestive heart failure caused by severe MR. This occurred 2 years after implantation of a transcatheter annuloplasty device (Carillon Mitral Contour System®) for presumed secondary MR at another institution. Coronary artery disease was excluded by coronary angiography. Following initial diuretic-driven decongestion, the patient was referred to our Heart Valve Center for further evaluation.

The patient was first diagnosed with severe secondary MR in 2019, in the context of paroxysmal atrial fibrillation and progressively worsening heart failure, initially managed with conservative therapy. Despite optimized, guideline-directed medical therapy, the patient remained symptomatic. Consequently, a Carillon device was implanted to address secondary MR through indirect annuloplasty, aiming to reduce the MR severity. While the patient initially reported symptomatic and quality-of-life improvement, progressive dyspnoea, peripheral oedema, and weight gain developed over subsequent year, ultimately leading to recurrent episodes of symptomatic heart failure.

**Figure 1:**
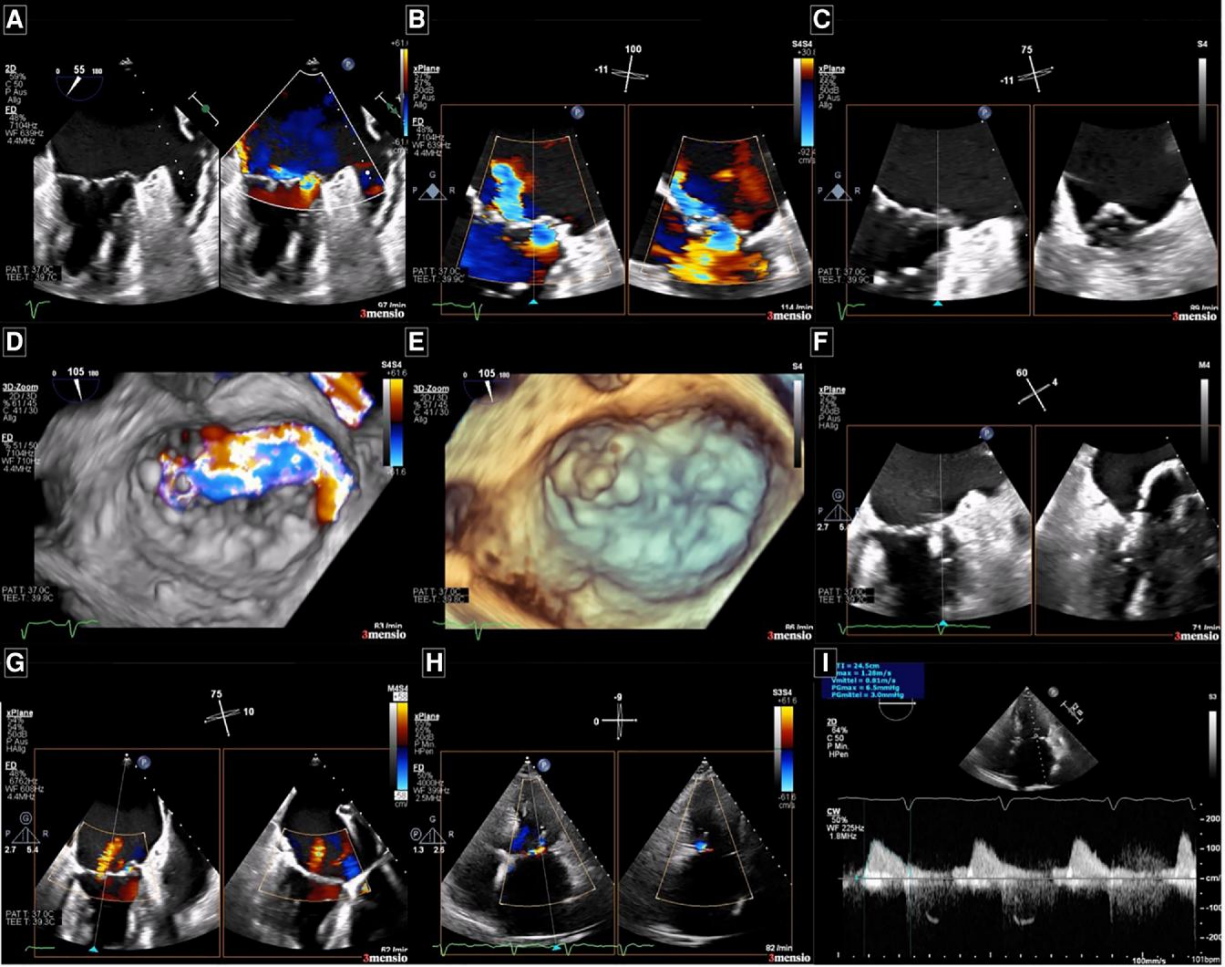
Diagnostic and intraprocedural echocardiographic imaging of the mitral valve before and after the first mitral transcatheter edge-to-edge repair procedure. (*A*) Mitral commissural view with simultaneous colour Doppler showing an eccentric regurgitant jet originating from the lateral commissure. (*B*) Zoomed biplane view through the lateral segments (A1/P1) of the mitral valve leaflets with colour Doppler. (*C*) Same biplane view without colour Doppler revealing a flail posterior mitral valve leaflet, the main pathology causing severe mitral regurgitation. (*D*) 3D en face view of the mitral valve during systole, with the aortic valve positioned at 12 o'clock, showing the eccentric jet originating from the A1/P1 segment. (*E*) 3D transoesophageal echocardiography image highlighting the flail posterior mitral valve leaflet responsible for the severe mitral regurgitation prior to MitraClip (Abbott®) placement. (*F*) Intraprocedural biplane imaging during device positioning targeting the lateral commissure. (*G*) Marked reduction in mitral regurgitation severity after initial clip deployment. (*H*) Post-procedural transthoracic echocardiography showing trace residual mitral regurgitation. (*I*) A mean mitral valve gradient of 3.0 mmHg was measured following the first clip implantation.

At our centre, transoesophageal echocardiography revealed mitral annular calcification involving the posterior ring and extending to the basal segment of the posterior mitral leaflet (PML), along with severe MR grade (4 out of 4) (*Figure 1A* and *B*; Supplementary material online, *Video S1A* and *B*). The regurgitation was attributed to posterior leaflet prolapse in combination with anterior leaflet flail, resulting in an eccentric jet (*Figure 1C–E*; Supplementary material online, *Video S1C–E*). Quantitative assessment demonstrated a proximal isovolumetric surface area [PISA] radius of 11 mm, a vena contracta width of 14 mm, and an effective regurgitation orifice area [EROA] of 0.8 cm^2^, and a regurgitant volume of 65 ml. Given the presence of annular calcification as a potential procedural challenge, the heart-team carefully evaluated therapeutic options and recommended M-TEER as the preferred approach, considering the patient’s age and elevated surgical risk (logistic EURO Score 16%).

For the initial procedure, a MitraClip® NTw (Abbott Medical) was implanted at the A1/P1 segment via transfemoral venous access using a 24F sheath and a transseptal puncture 4.5 cm above the mitral annulus. Device selection was guided by institutional experience and anatomical suitability at the time. Following three grasping attempts due to the complex anatomy, optimal device positioning and leaflet insertion at the lateral commissure were achieved (*Figure 1F*; Supplementary material online, *Video S1F*). MR was reduced to trace/mild (0/1+) without evidence of significant mitral stenosis (mean gradient 3 mmHg) (*Figure 1G–I*; Supplementary material online, *Video S1G*, H1, and H2). The post-procedural course was uneventful, and the patient was discharged 2 days after the intervention.

**Figure 2:**
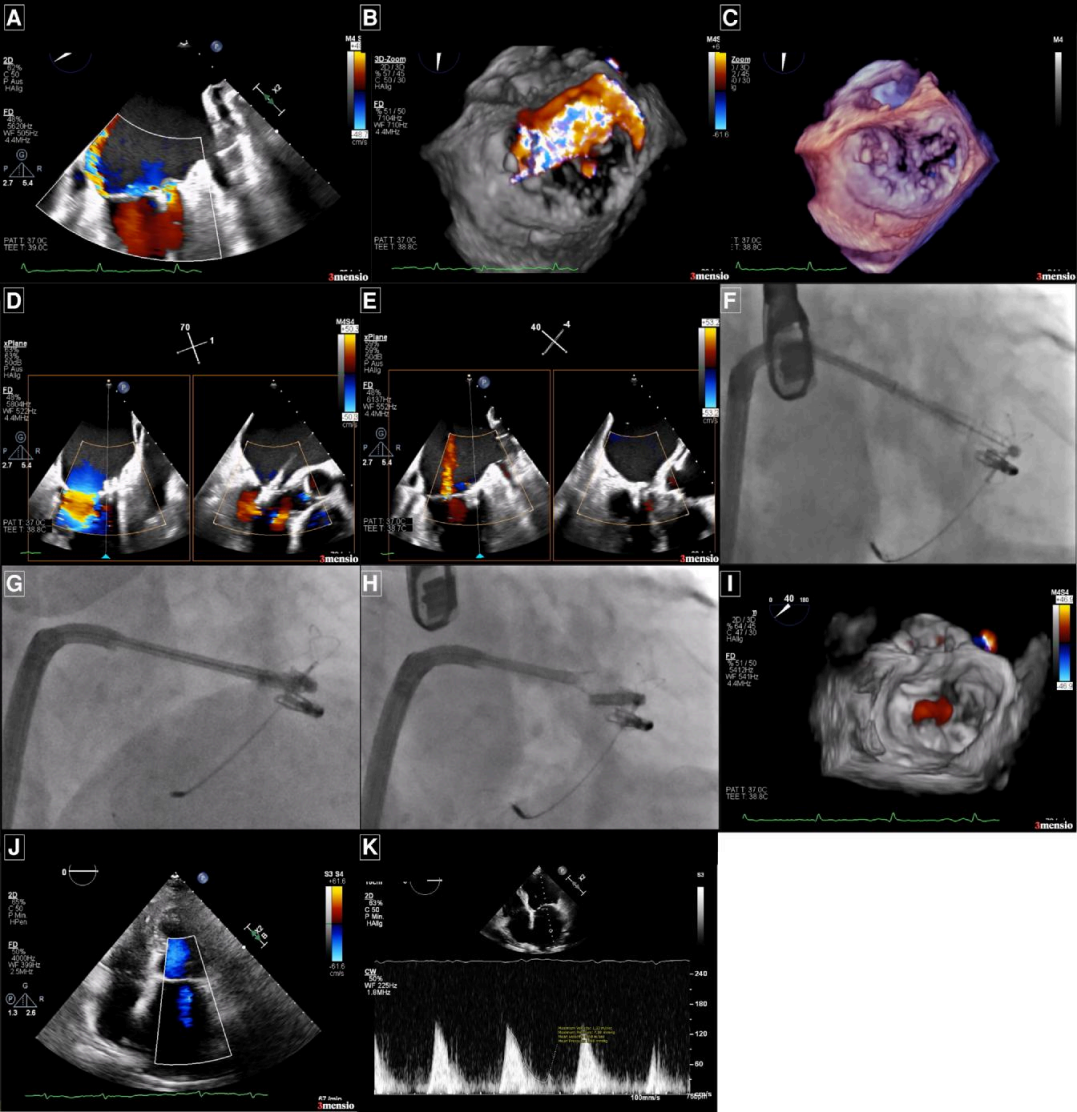
Diagnostic and intraprocedural echocardiographic and fluoroscopic imaging of the mitral valve before and after the second mitral transcatheter edge-to-edge repair procedure. (*A*) Mitral commissural view with colour Doppler showing the previously implanted clip at the A1/P1 segment, with a recurrent eccentric regurgitant jet originating from the lateral commissure. (*B*) 3D en face view of the mitral valve during systole, with the aortic valve at 12 o'clock, showing the eccentric jet arising lateral to the initial clip in the A1/P1 segment. (*C*) 3D transoesophageal echocardiography image revealing the residual prolapse of the posterior mitral valve leaflet responsible for the severe recurrent mitral regurgitation prior to the second mitral transcatheter edge-to-edge repair procedure. (*D*) Intraprocedural biplane imaging during device positioning with the PASCAL device (Edwards®), targeting the area between the first clip and the lateral commissure. (*E*) Demonstration of significant reduction in mitral regurgitation after deployment of the second device. (*F*, *G*, *H*) Fluoroscopic images capturing catheter approach to the target landing zone. (*I*) 3D view en face after second clip deployment. (*J*) Post-procedural transthoracic echocardiography showing only trace residual mitral regurgitation. (*K*) A mean transmitral gradient of 2.0 mmHg was recorded following the second device implantation.

Twenty-one months later, the patient presented to our structural heart disease outpatient clinic with recurrent symptoms (dyspnoea, enema), elevated NT-proBNP levels, and a recent heart failure hospitalization. Echocardiographic assessment was technically challenging due to acoustic shadowing from the previously implanted CARILLON® and MitraClip® devices, particularly impairing visualization of the lateral commissure of the mitral valve. Nevertheless, recurrent severe MR was confirmed, with two distinct regurgitant jets—one of which was an eccentric jet originating from the lateral commissure (PISA radius 13 mm) (*Figure 2A*; Supplementary material online, *Video S2A*). Visualization was optimized using transoesophageal multiplane imaging, complemented by 3D en-face reconstructions and deep transgastric views to assess all aspects of the valve, including both commissures. The pathology was attributed to residual prolapse of the calcified posterior leaflet, resulting in a 5 × 5 mm coaptation defect (*Figure 2B* and *C*; Supplementary material online, *Video S2B–D*). Severe tricuspid regurgitation was also noted, while left ventricular systolic function was preserved.

Following interdisciplinary heart team discussion, redo M-TEER was recommended. Ultrasound-guided venous access was obtained via a 22F sheath in the right femoral vein, and transseptal puncture was deliberately performed away from the prior puncture site to optimize for lateral trajectory. A PASCAL® Precision Ace device (Edwards Lifesciences) was advanced and elongated to access the remaining narrow orifice just lateral (*Figure 2D*; Supplementary material online, *Video S2E*) to the previously implanted MitraClip® at the lateral commissure (*Figure 2F–H*; Supplementary material online, *Video S2F* and *I*). Independent leaflet grasping was successfully achieved (*Figure 2I*; Supplementary material online, *Video S2J*) reducing MR to mild (+1) (*Figure 2E* and *J*; Supplementary material online, *Video S2K*). No significant mitral stenosis was observed (mean gradient 2 mmHg) (*Figure 2K*). Vascular access was closed using the ProStyle® closure system.

At one-year follow-up, echocardiography confirmed a stable result with maintained mild residual MR.

## Discussion

MR is broadly classified into degenerative and secondary types. DMR results from intrinsic pathology of the mitral valve leaflets or subvalvular apparatus, whereas secondary MR arises from left ventricular or atrial disease that impairs the function of an otherwise structurally normal valve. While surgery remains the gold standard for DMR, M-TEER has become an increasingly adopted alternative for DMR patients at high surgical risk, as reflected in current guideline recommendations,^2,3,6^

In this case report, the exact duration and progression of the patient’s MR and heart failure prior to referral remain unclear. The initial diagnosis in 2019 was presumed secondary MR, treated with the Carillon® Mitral Contour System (Cardiac Dimensions) following failure of optimal medical therapy.^8^ Upon evaluation at our centre, detailed echocardiographic assessment using a multiparametric approach revealed a severe calcified DMR as the dominant pathology.^9,10^ Although valve calcification was present, it can be difficult to detect with standard imaging modalities. On fluoroscopy, focal calcification may be obscured by the overlying spine, while echocardiography may underestimate the extent of calcification or fail to distinguish it from fibrosis. In this case, calcification did not preclude leaflet grasping but was carefully considered during device selection and procedural planning. Calcification can pose significant challenges during M-TEER by restricting leaflet mobility, reducing the effective grasping area, and increasing the risk of elevated post-procedural transmitral gradients. Nevertheless, given the patient’s advanced age and high surgical risk, transcatheter treatment was favoured over surgery.

Despite an initially successful outcome with M-TEER (residual MR 0/1+),^11^ the patient developed recurrent MR over the course of time. At 21-month post-intervention, severe recurrent MR was documented. Although M-TEER and surgical repair have demonstrated comparable mortality rates, surgical intervention is associated with greater durability in MR reduction.^5^ Approximately 10% of patients experience recurrent MR after an initially successful M-TEER.^7^ Redo M-TEER has emerged as a reasonable option for selected patients, particularly those with prohibitive surgical risk.^12^

Leaflet flail and prolapse, as observed in this case, are recognized mechanisms of recurrent MR and can progress over time—particularly when residual MR persists—even following technically successful leaflet approximation.^7,12^ However, most registry data focus on failure mechanisms such as single leaflet device attachment (SLDA) or complete loss of leaflet insertion.^12,13^ SLDA was not observed; however, device manipulation in the region of residual or recurrent regurgitation carries a risk of leaflet detachment or damage, particularly when probing with a second device to address prolapse adjacent to a prior implant. This underscores the importance of careful patient selection and meticulous procedural planning, and such complex interventions should be reserved for experienced centres and operators, respectively.

To address the complex lateral pathology and ensure precise positioning and manoeuvring during the redo procedure, the PASCAL Precision Ace® system was specifically selected. Its features—including a flexible nitinol frame, device elongation, and independent leaflet grasping—make it particularly well-suited for anatomically challenging cases, as demonstrated in the PASCAL IID registry.^14^ These characteristics facilitate precise navigation within narrow anatomical spaces and allow for secure capture.

In our view, the device enabled secure leaflet capture and effective MR reduction in this case, with a stable result and only mild residual MR observed at 1-year follow-up. The successful reduction of MR severity suggests that even in the presence of significant valve calcification and prior intervention, redo-M-TEER can be feasible when procedural strategies are carefully tailored to the individual anatomy and institutional experience.

From our perspective, this case illustrates the potential feasibility, safety, and durability of redo M-TEER using the PASCAL Precision Ace® system in the context of recurrent DMR due to progressive leaflet prolapse in the lateral commissure It highlights the importance of individualized anatomical evaluation, thoughtful device selection, and multidisciplinary heart-team decision-making, particularly in experienced, high-volume centres managing complex valvular heart disease in high-risk patients.

## Lead author biography


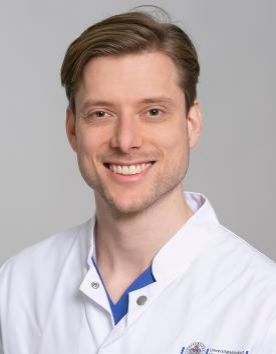


Cardiology Resident at the University Heart and Vascular Center Hamburg of the University Medical Center Hamburg Epppendorf since 2021.

## Supplementary Material

ytaf551_Supplementary_Data

## Data Availability

The data underlying this article will be shared on reasonable request to the corresponding author. Additional details from the patient´s medical history beyond those presented in this case report cannot be shared without explicit patient consent.
